# Editorial: Chemical and biological weapons

**DOI:** 10.3389/fmicb.2026.1907715

**Published:** 2026-07-17

**Authors:** Olli H. Tuovinen, Walter E. Grunden, Stephen A. Morse, Marion G. Dorsey, Lela Bakanidze

**Affiliations:** 1 Department of Microbiology, The Ohio State University, Columbus, OH, United States; 2 Department of History, Bowling Green State University, Bowling Green, OH, United States; 3 IHRC, Inc., Headquarters, Atlanta, GA, United States; 4 Department of History, University of New Hampshire, Durham, NH, United States; 5 National Center for Disease Control and Public Health, Tbilisi, Georgia

**Keywords:** biowarfare, chemical warfare, deterrence, pathogens, poisons, toxins

The year 2025 marked the centennial of the creation of the Geneva Protocol of 1925, which prohibited the use of chemical and biological weapons in warfare. The protocol emerged in the wake of World War I largely as a response to the use of poison gases (e.g., chlorine) by both the Central and Allied Powers beginning with the Second Battle of Ypres in 1915. World War I was the first conflict in which chemical weapons were used in mass quantities, and biological weapons were deployed in instances of sabotage, mainly being harnessed for use against animals, especially horses, upon which the ostensibly modern armies of the early twentieth century were still very dependent. While industrialization and the rise of modern chemistry made chemical warfare possible on a large scale, modern biological warfare was arguably still in its infancy, awaiting further development of the field of microbiology. Nonetheless, forward-looking delegates to the Geneva Convention foresaw the potential of biological weapons and argued for their inclusion in the prohibition as well. But to what extent can we say the Geneva Protocol was really effective? The Geneva Protocol banned only the *use* of chemical and bacteriological weapons in war. It did not forbid the research, production, or stockpiling of these weapons, which effectively created a massive loophole that allowed nations to amass huge arsenals. Despite such limitations, the Protocol established a vital, lasting moral and legal stigma against weapons of mass destruction. It promoted establishing a global norm, transforming the ban into customary international law that applies to all nations. Half a century later, the ban was expanded and reinforced by the Biological Weapons Convention (1975) and Chemical Weapons Convention (1997). Now, after 100 years, how shall we assess the efficacy of the Protocol?

These were questions on the minds of several authors involved in contributing to select editions of *Frontiers* to commemorate the centennial of the Geneva Protocol. The authors found it perplexing that so little attention was being paid to the 100-year anniversary of this landmark agreement and were determined to address the lacunae in historical and scientific literature covering this very important historical event.

Chemical and biological weapons were still used in varying degrees even after the ratification of the Geneva Protocol by many signatory nations and continued to be used even into the twenty-first century. No matter what this and other international agreements that followed proclaimed, powerful nation states and rogue adversaries were not entirely deterred. Today, there are not only those who use such weapons to gain dominance and control, but there are now bioterrorists who operate covertly to target specific individuals or groups. No international agreements seem able to deter such actors completely. Yet, too little attention has been paid to this subject matter outside the realm of government analysts and military historians.

The history of biological warfare dates back over a thousand years and incidents have been recorded in antiquity, such as using the carcasses or dead animals to spoil sources of fresh drinking water. But these were crude and unsophisticated methods, and more effective biological warfare would have to await the discovery of the germ theory of disease and the formation of the field of modern microbiology. The wars in Europe, especially in the mid- to late 1800s, prompted some of the first international treaties to address acceptable conduct in wartime, including non-conventional forms of warfare, such as those involving chemical and biological agents. By 1925, these two historical trends began to converge. Though the nineteenth-century treaties served as fundamental agreements and provided the foundation for subsequent treaties, continuous amendments were required to keep pace with scientific and technological advances, especially in fields having dual use applications for warfare.

Advances in chemical warfare seemed limited only by the pace of industrial development and production capacity, while the potential of unlimited microbial resources for warfare became apparent with studies published by French, German and British microbiologists at the turn of the twentieth century. The German Empire recruited and dispatched covert agents in World War I to carry pathogenic bacteria to Scandinavia, the United States, Argentina, Spain, and Romania. While the intended targets were predominantly war horses and mules, perhaps also sheep, agricultural targets, such as food crops, were also considered, though human targets were generally excluded at this time.

When the full impact and benefit of deterrence of unconventional weapons were recognized in the 1920s, their development, testing, and storage gained urgency among the developed nations. Although biological weapons were not deployed on a mass scale during World War II, perhaps out of concern for retaliation in kind, the testing of pathogenic bacteria with humans is now known to have been carried out by Germany and Japan, with the latter nation even engaging in limited use of biological agents against military forces of the Soviet Union and China, as well as civilian targets throughout their Asian empire. The Geneva Protocol of 1925 utterly failed to prevent these incidents of use, particularly as Japan refused to ratify the treaty until after World War II. The United States took even much longer −47 years.

The Cold War accelerated the research, development, and testing of chemical and biological warfare agents. This was an era of advanced experimentation, production, and stockpiling of mass quantities of these unconventional weapons. Perhaps predictably, accidents happened, and human lives were lost. More sophisticated analytical methods for detection and remediation were established, and R&D was initiated to develop physical, chemical and biological methods to detoxify and destroy old stocks even as new bioweapons and chemical pesticides were also being developed for use against agricultural crops for wartime situations.

With this historical context in mind, several *Frontiers* journal titles participated in the commemoration dedicated to the exploration of chemical and biological warfare and the impact of the Geneva Protocol treaty since its introduction 100 years ago. As this broad-spectrum topic is diverse and manifold, *Frontiers* recruited five special topic coeditors to expand the expertise of manuscript assessment for publication, including leading scholars in microbiology and history.

The published manuscripts cover areas ranging from the history of chemical and biological weapons to political and regional conflicts, education and analytics, biosecurity concerns, and the status to-date. Whereas, the centennial of the Geneva Protocol went largely unnoticed in other professional publications and venues, *Frontiers* took the lead in attempting to assess the impact and efficacy of the accord by featuring articles dedicated to this and related topics. Only through sustained analysis and dialogue can the true legacy of the Geneva Protocol of 1925 be determined. We thank all the contributing authors of the special topic and offer these articles in hopes of beginning that conversation.

